# Genome-Wide Identification, Expression Profiling, and microRNA397-Mediated Regulation of Laccase Genes in *Pinus massoniana*

**DOI:** 10.3390/plants15132032

**Published:** 2026-06-30

**Authors:** Guotao Song, Zhaoran Teng, Tengfei Shen, Wenlin Xu, Zihe Song, Meng Xu

**Affiliations:** State Key Laboratory of Tree Genetics and Breeding, Co-Innovation Center for Sustainable Forestry in Southern China, Nanjing Forestry University, Nanjing 210037, China; sgtendeavor@163.com (G.S.); zhaoran@njfu.edu.cn (Z.T.); tengfeishen@njfu.edu.cn (T.S.); wenlinxu@njfu.edu.cn (W.X.); zihesong@njfu.edu.cn (Z.S.)

**Keywords:** *Pinus massoniana* (Chinese red pine), *LACCASE* (*LAC*) gene family, microRNA397 (miR397)

## Abstract

Laccases (EC 1.10.3.2, LAC) are copper-containing glycoproteins involved in lignin biosynthesis, and as such, they play important roles in plant development and stress responses. In this study, a genome-wide analysis of the *LAC* gene family was performed in *Pinus massoniana* (Chinese red pine), identifying 78 *PmaLAC* genes, all predicted to encode cell membrane-localized proteins. These genes were unevenly distributed across eight chromosomes, with notable clusters on chromosomes 7 and 8, indicating gene duplication-driven expansion in *P. massoniana*. Phylogenetic analysis revealed that *PmaLAC* genes are classified into five subfamilies, reflecting the lineage-specific expansion and evolutionary divergence of gymnosperm *LAC* genes. Conserved motif and gene structure analyses showed high conservation among PmaLAC proteins. Promoter analysis identified numerous *cis*-acting elements related to hormone signaling, stress, and light responses. RNA-seq analysis revealed distinct tissue-specific expression patterns for *PmaLAC* gene family members. Moreover, degradome analysis combined with dual-luciferase assays supported the interaction between miR397c-9 and *PmaLAC31*, suggesting that miR397c-9 negatively regulates *PmaLAC31* and indicating a potentially conserved miRNA-mediated regulatory mechanism. Overall, this study provides a systematic overview of the composition, evolution, and potential regulation mechanisms of the *PmaLAC* gene family in *P. massoniana*, providing a useful resource for future functional characterization of *PmaLAC* genes.

## 1. Introduction

Laccases are multicopper oxidases (MCO) containing four copper ions and fall within the blue copper oxidase gene superfamily. They are extensively distributed among animals, plants, and microorganisms [1]. Laccases have attracted considerable attention because of their broad substrate specificity and diverse biological functions [1]. Laccases catalyze the oxidation of a wide variety of aromatic and non-aromatic compounds, including monophenols, diphenols, polyphenols, and polyamines [2]. The *LACCASE* (*LAC*) gene family is characterized by the presence of three conserved copper-oxidase domains, which constitute type I (T1), type II (T2), and type III (T3) domains [3]. Substrate oxidation occurs at the T1 site, whereas molecular oxygen is reduced at the T2/T3 trinuclear copper center [4]. *LAC* genes have been identified in numerous plant species, and according to the UniProt database [5], over 45,000 LAC proteins have been recorded from animals, plants, microorganisms, and fungi, with sequence lengths ranging from approximately 100 to 800 amino acids (aa).

Lignin is a major structural component of the plant secondary cell wall and a key determinant of vascular development [6]. Laccase, a central enzyme in lignin polymerization, regulates monolignol oxidation and coupling, thereby influencing plant growth, reproduction, and yield formation [7]. In *Arabidopsis thaliana* (Arabidopsis), the functions of *LACs* in growth and development have been well characterized. For instance, miR397b-mediated repression of *AthLAC4* expression significantly reduces lignin accumulation, resulting in increased seed size and seed number [8]. Furthermore, the *AthLAC2* mutant (*lac2* plants) exhibits shortened primary roots accompanied by excessive lignin deposition in vascular tissues [9], while the *AthLAC4 AthLAC11 AthLAC17* triple mutant (*lac4 lac11 lac17* plants) shows severe defects in vascular development and anther dehiscence, leading to growth arrest [10]. These studies demonstrate the essential roles of *LAC*s in vascular formation and reproductive development. *LAC*s and their miRNA regulators are also closely associated with crop yield formation. For example, in *Oryza sativa* (rice), overexpression of osa-miR397 enhances grain size and panicle branching by repressing *OsaLAC* gene expression, resulting in up to a 25% increase in yield under field conditions [11]. Also in *O. sativa*, *OsaLAC13* functions as a key regulator of reproductive development, with loss- and gain-of-function analyses revealing its roles in seed setting and fertility [12]. Similarly, miR408-mediated repression of *AtLAC13* in *A. thaliana* promotes biomass accumulation and seed yield [13]. Beyond growth and yield regulation, *LACs* contribute to fruit quality formation by modulating lignin composition. In *Citrus reticulata* (citrus), expression of a *LAC17*-like gene is associated with lignin monomer composition and fruit texture [14], while in *Pyrus bretschneideri* (Chinese white pear), several *PbrLAC* genes have been implicated in lignin biosynthesis and stone cell formation [15]. *LACs* also participate in plant responses to abiotic stresses and pathogen defense. Salt stress induces *ZmaLAC1* expression in *Zea mays* (maize), which is accompanied by altered root morphology [16]. In *Gossypium hirsutum* (cotton) and *Triticum aestivum* (wheat), *LAC*-mediated lignification has been shown to play critical roles in resistance to *Fusarium oxysporum* diseases, vascular wilt and Fusarium head blight, respectively [17,18]. Collectively, these studies indicate that the *LAC* gene family plays conserved and multifunctional roles in plant growth, development, lignification, environmental adaptation, and stress resistance.

*P. massoniana* is an ecologically and economically important evergreen conifer widely distributed in southern China and extensively used for reforestation, timber production, and industrial forestry [19]. Owing to its strong adaptability to arid and nutrient-poor soils, *P. massoniana* plays a crucial role in ecological restoration and sustainable forest management. In addition, its xylem is rich in cellulose for pulp and timber production, while its resin and secondary metabolites are valuable raw materials for pharmaceutical and chemical industries [20]. Despite its importance, the molecular mechanisms underlying wood formation, lignification, and stress adaptation in *P. massoniana* remain poorly understood. Although the *LAC* gene family has been extensively investigated in angiosperms, systematic studies in gymnosperms remain limited, particularly regarding miRNA-mediated regulatory mechanisms. This knowledge gap restricts our understanding of the evolutionary conservation and functional diversification of *LAC* genes in woody plants and conifers. Therefore, a comprehensive characterization of the *LAC* gene family in *P. massoniana* is of considerable significance for elucidating the molecular basis of lignification and adaptive development in gymnosperms [6,20]. In this study, we performed a genome-wide identification and comprehensive analysis of the *LAC* gene family in *P. massoniana*. We systematically characterized their phylogenetic relationships, conserved domains, gene structures, and motif compositions, and further constructed a putative miRNA-mediated regulatory network. In particular, the regulatory interaction between miR397c-9 and *PmaLAC31* was experimentally validated. Our findings provide new insights into the evolutionary and functional characteristics of *LAC* genes in gymnosperms and offer a basis for further investigation of lignin-related regulatory mechanisms in *P. massoniana*.

## 2. Materials and Methods

### 2.1. Plant Materials

Plant samples were obtained from superior clonal lines of *P. massoniana* cultivated in Guangxi Province, China (106°30′–107°34′ E, 21°46′–22°03′ N). Three grafted ramets from ten-year-old individuals were selected as independent biological replicates. To investigate tissue-dependent expression profiles, six types of tissues were harvested from the three biological replicates, including immature female strobili (PF), immature male strobili (SF), immature cones at 12 months (BF), one-year-old needles (NL), young stems at three months of age (TS), and bark tissues (TB), which consisted of phloem and developing xylem [21]. All samples were immediately harvested and stored in liquid nitrogen and then transferred to −80 °C.

### 2.2. Sequence Alignment and Homolog Identification

Genomic resources of *P. massoniana*, including the assembled genome, annotated gene models, and protein sequences, were retrieved from the GIGA database [22]. The downloaded genome was subsequently re-annotated to improve gene model accuracy and facilitate downstream analyses. In addition, in-house transcriptome and degradome sequencing data were generated to support subsequent genomic analyses [21], including 18 transcriptome libraries from six tissues and one degradome library constructed by pooling equal amounts of total RNA (2 µg per tissue) from the same tissues.

To ensure accurate and comprehensive identification of *LAC* genes in *P. massoniana*, a genome-wide screening strategy integrating two independent approaches was employed [23]. First, three hidden Markov model (HMM) profiles corresponding to conserved MCO domains (PF07732, PF00394, and PF07731) were obtained from the InterPro database [24]. These profiles were used to search the *P. massoniana* proteome with HMMER v3.4 [25], applying a stringent E-value criterion of 1 × e^−20^ [26]. Second, BLASTP v2.16.0 [27] analysis was performed against the *P. massoniana* proteome using AthLAC proteins as queries, with a cutoff value of 1 × e^−5^ [28]. Candidate genes identified by both strategies were filtered through TBtools v2.472 [29] and regarded as high-confidence *PmaLAC* candidates.

To further verify candidate LAC protein reliability, the presence of conserved domains was verified using NCBI Conserved Domains Database (CDD) [30]. Subsequently, ExPASy ProtParam was employed to calculate molecular features [31]. Protein subcellular localization was predicted using Cell-PLoc v2.0 [32], while SignalP v6.0 [33] and TMHMM v2.0 [34] were used for signal peptide and transmembrane helix prediction.

### 2.3. Phylogenetic Reconstruction and Conserved Domains

To investigate the evolutionary relationships and classification of LAC proteins, a phylogenetic analysis was conducted using LAC protein sequences from the gymnosperm *P. massoniana* and the representative angiosperms *A. thaliana* and *Populus trichocarpa* (black cottonwood) [35]. Protein sequences were first aligned using ClustalW v2.1 [36] with default parameters to generate a multiple sequence alignment. Based on the aligned sequences, a maximum likelihood (ML) phylogenetic tree was constructed using RAxML v8.2.12 [37]. The JTT amino acid substitution model with Gamma-distributed rate heterogeneity (PROTGAMMAJTT) was employed, and branch reliability was assessed by performing 1000 bootstrap replicates. The resulting phylogenetic tree was visualized and annotated using iTOL v7.0 to facilitate clade classification and comparative analysis [38].

To further examine structural conservation among PmaLAC proteins, conserved domains were identified using Batch CD-Search against the CDD [30], and only superfamily-level annotations were retained. Meanwhile, conserved motifs were predicted using the MEME Suite [39], under the Zero or One Occurrence Per Sequence (ZOOPS) model with a limit of ten motifs.

### 2.4. Gene Architecture and Promoter cis-Element Profiling

To characterize the genomic organization of *PmaLAC* genes, gene structure (exon–intron organization) and chromosomal positions were retrieved from the genome annotation data using TBtools [29], and the data were integrated for visualization.

To explore the potential transcriptional regulation of *PmaLAC* genes, promoter regions were analyzed for *cis*-acting regulatory elements. Specifically, 2000 bp upstream sequences relative to the translation start codon (ATG) were extracted as putative promoter regions using samtools v1.23.1, taking gene strand information into account [40]. The extracted promoter region sequences were analyzed using the PlantCARE database [41] for *cis*-element identification. Following curation of the results, the distribution patterns of *cis*-acting elements were visualized using ggplot2 v4.03 [42] in R v4.5.2.

### 2.5. Synteny Analysis Using MCScanX

To investigate the duplication patterns and evolutionary expansion of the *PmaLAC* gene family, genomic collinearity and duplication events were analyzed using MCScanX v1.0.0 [43]. Protein sequences from the complete genomes of *P. massoniana* and the closely related conifer species *P. tabuliformis* were subjected to all-versus-all BLASTP analysis using BLAST v2.16.0 with an E-value cutoff of 1 × e^−5^. Based on the BLAST results, MCScanX was employed to identify syntenic blocks and classify gene duplication events, including the distinction between segmental and tandem duplication events. *PmaLAC* gene pairs located within conserved syntenic regions were subsequently extracted to infer the evolutionary divergence of duplicated genes. The genomic collinearity relationships among *PmaLAC* genes were visualized using Circos v0.69 [44] to facilitate comparative interpretation of duplication patterns.

### 2.6. TPM-Based Analysis of PmaLAC Gene Expression Patterns

To investigate tissue- and development-specific expression patterns of *PmaLAC* genes, an in-house RNA-seq dataset comprising six tissue types (each with three biological replicates per sampled tissue) was used. Following quality assessment, one sample (TB3) failed library preparation and was excluded from further analyses. Consequently, the final dataset consisted of 17 high-quality samples, with two biological replicates retained for the TB tissue and three biological replicates for the remaining tissues. Quality control and trimming of paired-end reads were performed with fastp v0.21.0 [27] prior to downstream analyses. The filtered reads were then aligned to the *P. massoniana* reference genome using HISAT2 v2.2.1 [45] with a pre-built genome index. Gene-level read counts were obtained using featureCounts v2.0.8 [46]. Read counts were normalized to Transcripts Per Million (TPM) using R v4.5.2, and the mean TPM values across biological replicates for each tissue were calculated for cross-sample comparisons. Subsequently, hierarchical clustering heatmaps were generated from log_2_^(TPM+1)^, while Z-score normalized TPM values were used to plot expression trend line graphs, with genes grouped by cluster to show tissue- and stage-specific patterns of expression for each identified *PmaLAC* gene.

### 2.7. Prediction of miRNA-Binding Sites

To investigate potential regulatory interactions between miR397 and *PmaLAC* genes, miRNA prediction and target identification were performed based on the *P. massoniana* genome, sRNA-seq, and degradome sequencing data. Candidate miR397 and its precursor sequence were predicted using sRNAminer v1.1.2 [47] with default parameters. Degradome sequencing data from six tissues were subjected to quality control and adapter trimming using fastp v0.21.0 [27] prior to downstream analysis (Table S11). High-quality degradome reads were then integrated with the predicted miR397 mature sequences and *PmaLAC* cDNA sequences to identify miRNA-mediated cleavage sites using CleaveLand4 v4.5 [48]. Only interactions with category ≤ 2 were retained for subsequent Target plot (T-plot) analysis, and the regulatory network between *PmaLAC* genes and miR397 was constructed and visualized using Cytoscape v3.6.0 [49].

### 2.8. Dual Luciferase Reporter Assay for Validating miRNA-Target Interactions

To functionally validate the miR397c-9-*PmaLAC31* regulatory relationship, a dual-luciferase reporter assay was conducted as previously described [50]. To perform the assay, the *PmaLAC31* open reading frame (ORF) was cloned downstream of the luciferase reporter gene in the pGreenII 0800-miRNA vector, while the miR397c-9 precursor (pre-miR397c-9) was inserted into the pGreenII 62SK vector for overexpression. To inhibit miR397c-9 activity, a short tandem target mimic (STTM397c-9) construct was also generated in the pGreenII 62SK vector [51]. Each construct was individually transformed into *Agrobacterium tumefaciens* [52]. Bacterial suspensions (OD_600_ ≈ 1.0) harboring different plasmid combinations were co-infiltrated into the abaxial side of *Nicotiana benthamiana* following selection of positive colonies. Firefly (Fluc) and Renilla (Rluc) luciferase activities were measured 48 h post-infiltration using a GloMax^®^ 96 microplate luminometer (Madison, WI, USA). Regulatory activity was evaluated based on Fluc/Rluc ratios normalized to the control.

To further validate the regulatory effect in a native system, the pre-miR397c-9 and STTM397c-9 sequences were cloned into the pJIT166 plant expression vector used for transient overexpression of inserted sequences, and the resulting constructs were transfected into *P. massoniana* protoplasts. Total RNA was extracted from the protoplasts 16 h post-transformation, followed by cDNA synthesis. Quantitative reverse transcriptase PCR (RT-qPCR) was then performed to quantify the expression levels of the miR397c-9 target gene, *PmaLAC31*. The specific procedures for the protoplast isolation and transient transformation were performed according to our previous study in our laboratory [53].

All dual-luciferase assays and protoplast transient transformation experiments were independently performed three times. For the dual-luciferase assay, three infiltrated leaves from each *N. benthamiana* plant were collected for luciferase activity measurements, and the average Fluc/Rluc ratio was used to evaluate regulatory activity. Changes in *PmaLAC31* expression in transformed protoplasts were determined by RT-qPCR. Data are presented as mean ± SD, and statistical significance was evaluated using Student’s *t*-test, with *p* < 0.05 considered statistically significant.

## 3. Results

### 3.1. Identification of LAC Genes in P. massoniana

A genome-wide analysis of *P. massoniana* identified 78 *PmaLAC* genes. The predicted proteins ranged from 539 to 638 aa in length, with an average of 575 aa, and molecular weights varied between 59.57 and 71.20 kDa. Isoelectric points spanned 5.33 to 9.59, with approximately 68% of the proteins being basic (pI > 7), suggesting that most PmaLAC proteins may maintain stability under specific physiological pH conditions. The aliphatic index ranged from 74.57 to 90.87, and around 94% of the predicted proteins exhibited a negative GRAVY value (−0.24 to 0.04), indicating a predominance of hydrophilicity. Subcellular localization prediction using Cell-PLoc 2.0 indicated that all PmaLACs are cell membrane-localized proteins (Table S1). SignalP and TMHMM analyses showed that most PmaLAC proteins harbor a predicted N-terminal signal peptide and one transmembrane helix, whereas some other (n = 9) family members were classified as “OTHER”, lacking clear signal peptides or transmembrane regions (Table S2). Overall, the PmaLAC protein family exhibited variation in length and physicochemical properties, reflecting structural diversity within the family.

### 3.2. Phylogenetic Relationships and Conserved Motif Analysis

**Figure 1:**
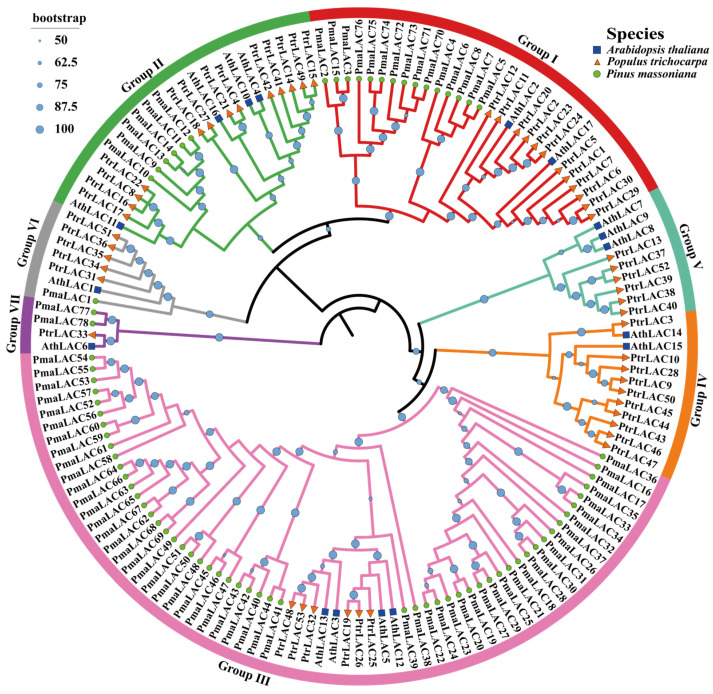
Phylogenetic analysis across multiple species. In the figure, the three different shapes represent three different species: blue squares for *Arabidopsis thaliana*, yellow pentagrams for *Populus trichocarpa*, and green circles for *Pinus massoniana*. Branches with different colors and the outermost ring in different colors represent distinct subfamilies, with subfamily names assigned based on the classification in *Arabidopsis thaliana*. Light blue circles on different branches indicate bootstrap support values ranging from 50 to 100, and larger circles represent higher bootstrap support values.

Comparative analyses of LAC protein sequences from *A. thaliana* (17 AthLACs), *P. trichocarpa* (53 PtrLACs), and *P. massoniana* (78 PmaLACs) were conducted to elucidate their evolutionary relationships (Figure 1). Phylogenetic analysis classified AthLAC and PtrLAC proteins into seven distinct subgroups (Groups I–VII), whereas PmaLAC proteins were distributed among only five of the seven groups identified. Specifically, Group I comprised 15 PmaLACs, Group II contained six PmaLACs, and Group III represented the largest subgroup with 54 members. In contrast, only one and two PmaLACs were assigned to Groups VI and VII, respectively, and no gymnosperm LACs were identified in Groups IV or V (Figure S1A). Distinct subgroup distribution patterns were observed between gymnosperms and angiosperms. Gymnosperm LACs were predominantly enriched in Groups I and III, exhibiting markedly higher gene numbers than those in angiosperms, whereas the sizes of Groups II, VI, and VII were relatively comparable between the two lineages.

Conserved motif analysis further corroborated the structural conservation of the PmaLAC proteins (Figure S1B), as most family members retained all ten identified motifs, while motif loss was detected in only a limited number of genes. Specifically, PmaLAC12 and PmaLAC13 lacked motif 7, whereas PmaLAC9, PmaLAC10, PmaLAC11, PmaLAC14, and PmaLAC40–PmaLAC44 were missing motif 8. The remaining motifs were consistently present and displayed uniform positional arrangements across the body of each identified PmaLAC protein.

### 3.3. Gene Structure and Promoter Region Element Analysis

A systematic analysis of gene structure was conducted for the *PmaLAC* family in *P. massoniana*. The 78 *PmaLAC* genes exhibited considerable variation in gene length, ranging from 2.4 to 8.2 kilobases (kb), with *PmaLAC58* being the longest and *PmaLAC12* the shortest members of the family (Figure S1C). Variation in gene length was primarily attributable to differences in intron size. All *PmaLAC* genes contained six or more introns. Integration of gene structure data with the maximum-likelihood phylogenetic tree showed that *PmaLAC* genes within the same clade generally shared similar exon–intron organization and conserved motif arrangement (Figure S1A).

**Figure 2:**
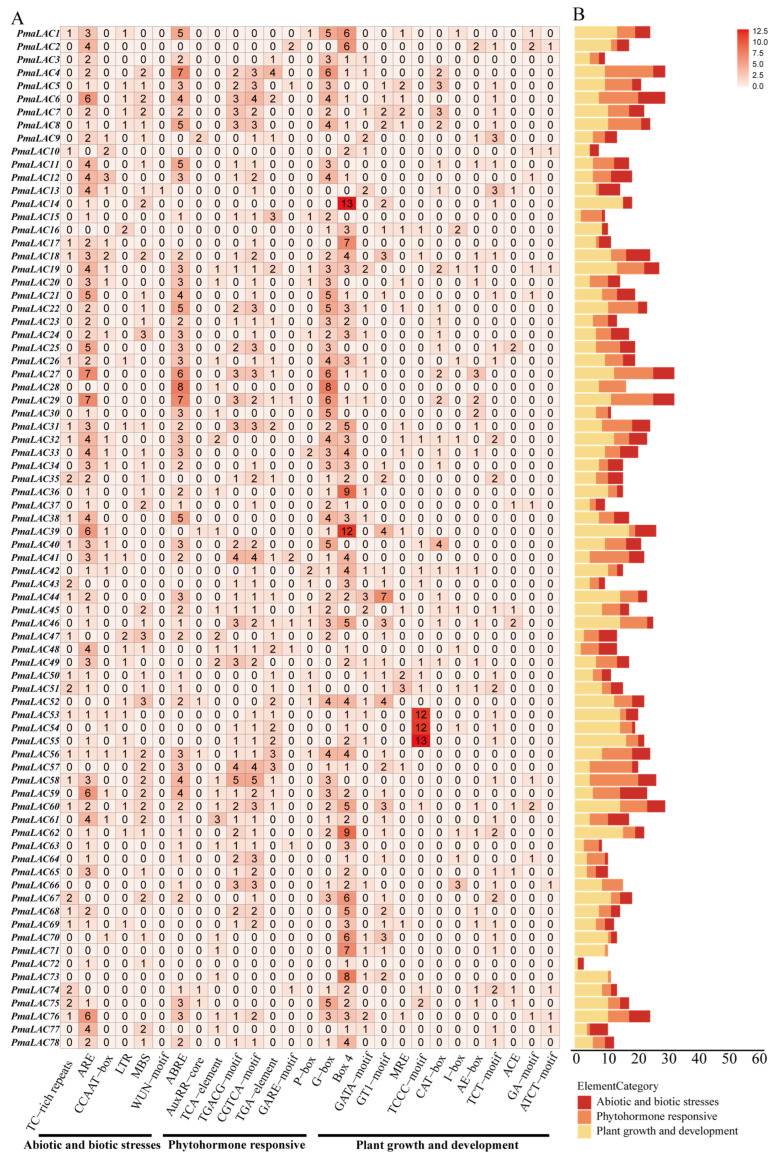
Heatmap of *cis*-acting elements in *PmaLAC* promoters. (**A**) Abundance of different cis-acting elements in *PmaLAC* promoters, with darker colors indicating higher numbers of elements. (**B**) Statistical summary of the numbers of cis-acting elements across three different functional categories.

A total of 27 *cis*-acting elements were identified and grouped into three functional categories: phytohormone-responsive, abiotic and biotic stress-related, and plant growth and development-related (Figure 2A). The highest abundance was observed for *cis*-elements associated with growth and development among these categories, accounting for more than half of all identified *cis*-elements, followed by hormone-responsive elements, whereas stress-related elements were comparatively less frequent (Figure 2B). Several key regulatory motifs, including the TGACG-motif, the CGTCA-motif, the ABRE and the TCA-element, were widely distributed across the *PmaLAC* promoter regions. Except for *PmaLAC71* and *PmaLAC73*, which lacked stress-responsive elements, and *PmaLAC10*, *PmaLAC14*, and *PmaLAC16*, which lacked hormone-responsive elements, all other *PmaLAC* genes contained *cis*-elements from all three categories.

### 3.4. Genomic Location and Gene Duplication Events

**Figure 3:**
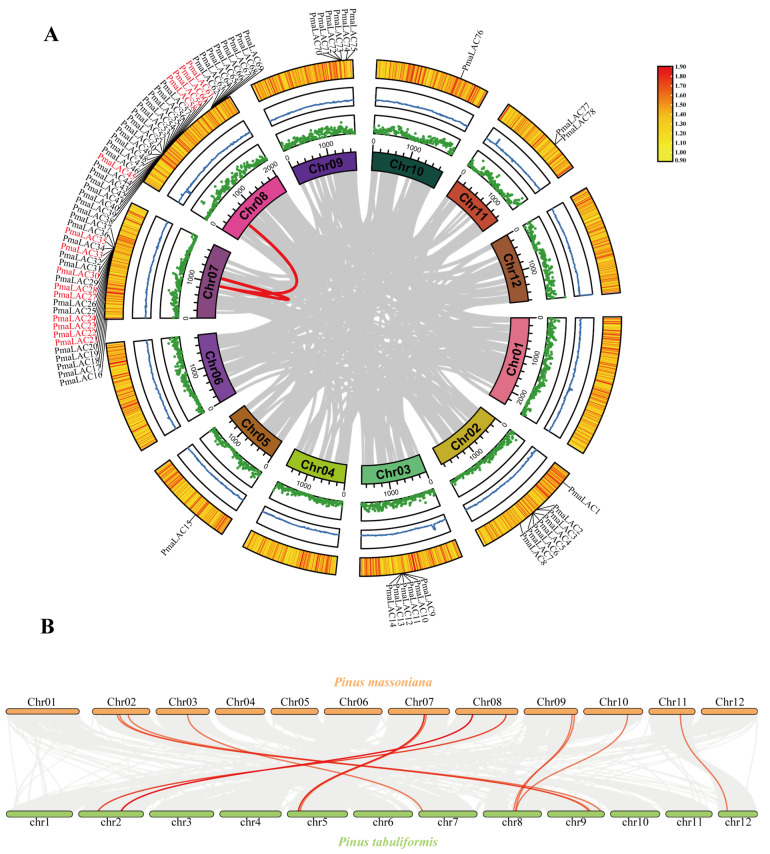
Synteny analysis of *LAC* gene pairs. (**A**) Chromosomal synteny analysis of the *PmaLAC* gene family. All *PmaLAC* genes were mapped onto chromosomes, and the identified syntenic *PmaLAC* gene pairs are highlighted in red and connected by red lines. From the innermost to the outermost circle, the tracks represent chromosome positions, proportion of undetermined bases (N ratio), GC content ratio (GC ratio), and gene density. Several synteny links and gene clusters are observed on Chr. 7 and Chr. 8, suggesting these regions may serve as duplication hotspots. (**B**) Synteny analysis of *LAC* genes between *Pinus massoniana* and *Pinus tabuliformis*. Chromosomes from different species are distinguished by different colors, and syntenic gene pairs are linked by red lines. revealing a high level of chromosomal collinearity between the two species.

Chromosomal localization analysis was conducted for the 78 *PmaLAC* genes in the *P. massoniana* genome. As depicted in Figure 3A, the distribution of these genes was uneven, spanning eight out of the twelve chromosomes. More than half of the *PmaLAC* genes were concentrated on Chr. 7 (22 genes) and Chr. 8 (32 genes), with Chr. 8 harboring the largest number of family members, likely due to the presence of prominent gene clusters. In contrast, only four *PmaLAC* genes were located on Chrs. 5, 10, and 11, each existing as an isolated single-gene locus. In addition, several chromosomes (Chrs. 2, 8, and 9) contained two or more spatially separated clustering regions (Figure S2).

In the intraspecific synteny analysis of the *P. massoniana* genome, extensive homologous blocks were detected across multiple chromosomes (Figure 3A). Notably, prominent synteny hotspots were observed on Chr. 7 and Chr. 8. A total of 15 syntenic relationships involving *PmaLAC* genes (involving 14 *PmaLAC* genes) were identified across all 12 chromosomes (Table S3). Among these duplicated genes, *PmaLAC21*, *PmaLAC22*, *PmaLAC23*, *PmaLAC24*, *PmaLAC33*, and *PmaLAC35* formed prominent gene clusters that extensively overlapped with syntenic links. Furthermore, gene density analysis revealed relatively high gene densities within these synteny hotspots.

Comparative synteny analysis between *P. massoniana* and *P. tabuliformis* revealed a high degree of overall chromosomal collinearity (Figure 3B). Among the 78 *PmaLAC* genes identified, 18 family members formed stable orthologous gene pairs between the two species.

### 3.5. Expression Analysis of PmaLAC Gene Family Members

Transcriptome data from six tissues of *P. massoniana* was analyzed to characterize the expression profiles of the 78 identified *PmaLAC* genes (Table S4). Hierarchical clustering and heatmap visualization revealed pronounced tissue-specific expression patterns (Figure S3A). However, the expression of neither *PmaLAC32* nor *PmaLAC50* could be detected in any of the sampled tissues. In direct contrast, the remaining 76 genes exhibited substantial expression variation across tissues, indicating strong tissue specificity (Table S5). Most *PmaLAC* genes were primarily expressed in one or two tissues or displayed peak expression in a specific tissue.

The expression trend clustering plot was based on Z-score-normalized TPM values. Figure S3B shows that the *PmaLAC* genes were classified into eight distinct clusters (Clusters 1–8). Cluster 3 (20 genes) and Cluster 7 (18 genes) were the largest groups, exhibiting relatively broad expression across multiple tissues. In contrast, Cluster 5 (2 genes) and Cluster 8 (3 genes) contained the smallest number of family members and displayed pronounced tissue-specific expression (Table S6). Cluster-specific preferences were observed: genes in Clusters 2, 4, 5, 6, and 8 were predominantly expressed in young stems (TS), bark (TB), male strobili (SF), female strobili (PF), and needles (NL), respectively. Notably, transcripts of nearly all *PmaLAC* genes were detectable in female strobili.

### 3.6. pma-miR397–PmaLAC Regulatory Modules in P. massoniana

miRNA prediction based on sRNA-seq was performed using sRNAminer (Table S7), which resulted in the identification of 34 miR397 family members. Although their precursor sequences were distinct, the mature sequences could be grouped into eight types, with one being 20 nt in length and the remainder 21 nt (Table S8). Phylogenetic analysis of both precursor and mature sequences revealed that gymnosperms exhibited a distinct evolutionary pattern compared with angiosperms, mainly characterized by a larger number of family members (Figure S4A), while their mature sequences retained a highly conserved 14 bp core region (Figure S4B). Expression analysis based on sRNA-seq data showed obvious expression divergence among different subfamilies, whereas members within the same subfamily displayed similar expression patterns. Among them, members of the miR397a, miR397c, and miR397g subfamilies exhibited high expression levels across all six tissues (Figure S5; Table S9). The promoters of *MIR397* genes contained various cis-acting elements, among which MYB-binding elements were significantly enriched (Figure S6). Degradome analysis using CleaveLand4 identified 21 miR397 members targeting 43 *PmaLAC* genes, resulting in a total of 70 pma-miR397–*PmaLAC* interactions (Figure S7; Tables S10–S12). Among these interactions, 21 pairs were supported by strong degradome cleavage singles and were classified as high-confidence interactions (Category ≤ 2) (Figure S8), whereas the remaining pairs belonged to Categories 3–4. These results suggest that miR397, a highly conserved and functionally well-characterized laccase-regulating miRNA in woody plants, may serve as the major regulator of *PmaLAC* genes.

### 3.7. pma-miR397c-9-Mediated Repression of PmaLAC31 Expression

Based on our in-house degradome data, together with the identified members of the miR397 family, CleaveLand4 was used to identify mRNAs targeted by miR397. The analysis revealed that pma-miR397c-9 can suppress *PmaLAC31* expression via a cleavage-based mode of RNA silencing, with a degradome Category of 0 and a statistically significant *p*-value of 0.0111, indicating a highly reliable cleavage event (Figure S8). Furthermore, pma-miR397c-9 exhibited high sequence complementarity with *PmaLAC31*, supporting a regulatory relationship between the miRNA and this *LAC* gene.

**Figure 4:**
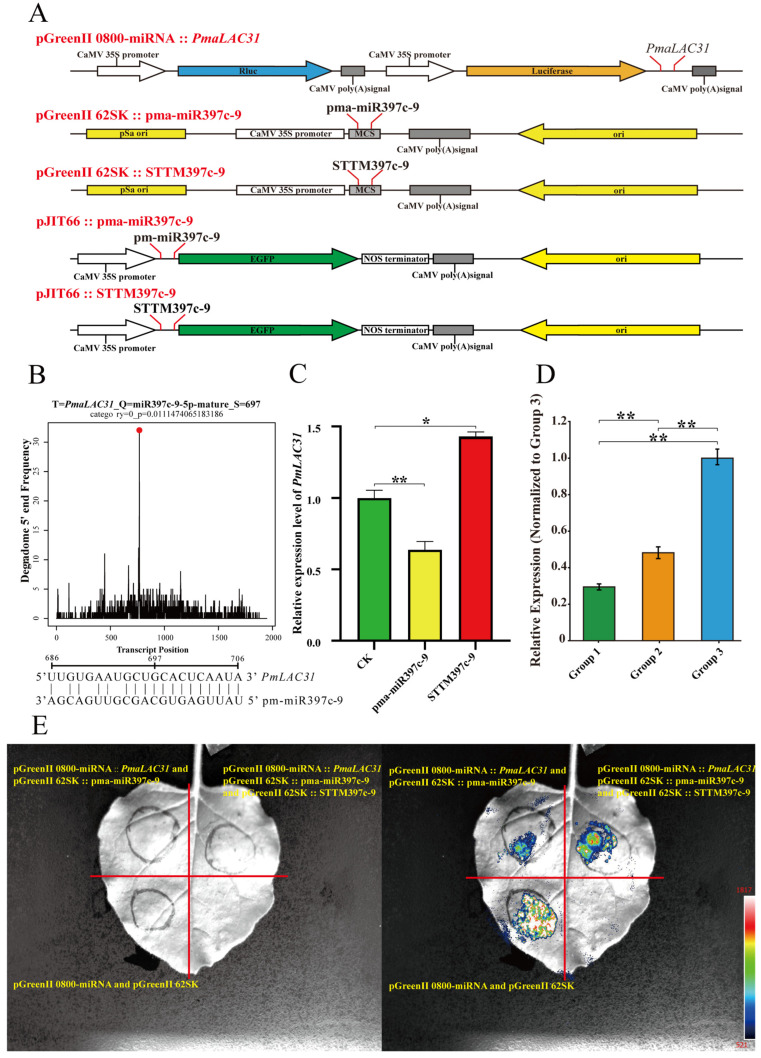
Experimental validation of the pma-miR397c-9-*PmaLAC31* regulatory module. (**A**) Schematic representation of the dual-luciferase reporter and pJIT166-based transient overexpression vectors, illustrating the specific cloning sites for pma-miR397c-9, STTM397c-9, and the *PmaLAC31* target sequence. (**B**) T-plot derived from degradome sequencing, identifying the precise cleavage sites of representative pma-miRNAs on the *PmaLAC31* transcript. (**C**) Relative expression levels of *PmaLAC31* in different *Pinus. massoniana* protoplast treatment groups, calculated using the 2^−ΔΔt^ method. including the empty-vector control (CK, normalized to 1.0), the pma-miR397c-9 overexpression group, and the STTM397c-9 group. Asterisks indicate significant differences based on Student’s *t-*test (* *p* < 0.05, ** *p* < 0.01). (**D**) Normalized fluorescence ratios (LUC/REN) were measured using a microplate reader across three experimental groups: Group 1 (pGreenII 0800-miRNA::*PmaLAC31* + pGreenII 62SK::pma-miR397c-9), Group 2 (pGreenII 0800-miRNA::*PmaLAC31* + pGreenII 62SK::pma-miR397c-9 + pGreenII 62SK::STTM397c-9), and Group 3 (empty-vector control: pGreenII 0800-miRNA + pGreenII 62SK). Double asterisks indicate statistically significant differences based on Student’s *t*-test (** *p* < 0.01). (**E**) Chemiluminescence imaging of co-transformed *Nicotiana benthamiana* leaves (left: bright-field/grayscale reference; right: bioluminescence overlay), visually demonstrating the in vivo repression of the target gene by miR397c-9.

To experimentally validate this regulatory relationship, *PmaLAC31*, pma-miR397c-9, and STTM397c-9 sequences (Figure S9) were cloned into dual-luciferase reporter vectors and the pJIT166 expression vectors, respectively (Figure 4A,B and Figures S10–S12; Table S13). Subsequently, a protoplast transient expression system derived from *P. massoniana* tissues was employed to investigate the regulatory interaction. RT-qPCR analysis showed that, compared with the control group transformed with the empty pJIT166 vector, the transcript level of *PmaLAC31* was significantly reduced following overexpression of pma-miR397c-9. In contrast, inhibition of endogenous miRNA activity via STTM397c-9 resulted in a detectable increase in *PmaLAC31* expression relative to the control (Figure 4C). In planta, a transient co-expression assay using a dual-luciferase reporter system was conducted in *N. benthamiana* leaves. Quantitative luciferase assays showed that, relative to the empty-vector control (Group 3, normalized to 1.0), co-expression of pma-miR397c-9 and *PmaLAC31* (Group 1) led to an approximately 70% decrease in luciferase activity, with the relative value reduced to ~0.29 (*p* < 0.01), suggesting that pma-miR397c-9 exerts a suppressive effect on *PmaLAC31* expression (Figure 4D). In contrast, co-expression of the miRNA inhibitor STTM397c-9 in a three-component system (Group 2) partially alleviated this repression, restoring luciferase activity to ~0.48 (*p* < 0.01), although it remained lower than that of the empty-vector control (Figure 4D). Consistent with these findings, in vivo chemiluminescence imaging further supported the quantitative results (Figure 4E). The region infiltrated with the target gene and miRNA alone (top-left) exhibited a weak luminescent signal, whereas co-expression with STTM397c-9 (top-right) resulted in a noticeable recovery in both signal intensity and spatial distribution.

These complementary results provide additional evidence supporting the targeting and cleavage of *PmaLAC31* by pma-miR397c-9. All experiments were independently performed in triplicate and yielded consistent results, indicating the robustness and reproducibility of this regulatory interaction. Collectively, these findings suggest that miR397c-9 contributes to the regulation of *PmaLAC31* expression.

## 4. Discussion

Members of the *LAC* gene family are widely distributed in both prokaryotes and eukaryotes and represent an important member of the MCO gene superfamily [54]. Specifically, LAC proteins have been shown to play crucial roles in lignin biosynthesis [55], cell wall formation, plant growth and development, as well as mediating responses to abiotic stresses [56]. While extensively characterized in model plants, the *LAC* gene family remains much less well characterized in the economically and ecologically important gymnosperm *P. massoniana*.

In this study, a total of 78 *LAC* genes were identified in the *P. massoniana* genome. The size of the *PmaLAC* gene family is comparable to that reported in other woody plant species, such as *P. trichocarpa* (53 members) [57] and *P. densiflora* (54 members) [58], but is markedly larger than those observed in herbaceous and graminaceous species, including *A. thaliana* (17 members) [3] and *O. sativa* (30 members) [59]. The expansion of this gene family in woody plants may be associated with their greater reliance on lignin biosynthesis and secondary cell wall formation. In addition, most PmaLAC proteins were predicted to contain an N-terminal signal peptide and a transmembrane helix, suggesting potential secretion-related functions. Gene Ontology (GO) annotation analysis of *PmaLAC* genes showed significant enrichment in oxidoreductase activity and response to copper ion (Figure S13; Table S14). This is consistent with laccases being multicopper oxidases, suggesting their key roles in metal ion regulation and redox metabolism in *P. massoniana* [1].

Phylogenetic analysis further revealed that *LAC* genes from herbaceous *A. thaliana* and angiosperm *P. trichocarpa* can be classified into seven subfamilies, whereas *PmaLAC* genes are distributed into only five relatively ancient subfamilies [1], suggesting that the *LAC* gene family has undergone lineage-specific expansion and contraction during plant genome evolution [60]. A few nodes with bootstrap values <50 were mainly located at deep divergence points among major clades, likely due to high sequence conservation and gene family expansion in *P. massoniana* and other plants, which reduces phylogenetic resolution at deeper evolutionary levels [61]. Notably, *PmaLAC* genes are predominantly enriched in specific subfamilies (Groups I and III), while being absent in others (Groups IV and V), suggesting a biased expansion pattern in gymnosperms. This pattern may reflect the functional specialization of specific *LAC* gene subclades in response to the unique requirements of conifer wood formation. Conifers are characterized by extensive secondary wall growth [62], lignin-rich wood [63], and a predominance of guaiacyl (G-type) lignin units [64], which differ from the more diverse lignin composition found in angiosperms. The expansion of specific *PmaLAC* subfamilies may therefore be associated with increased demand for lignin polymerization and structural reinforcement in long-lived woody tissues. In particular, the enrichment of *PmaLACs* in Groups I and III may contribute to the regulation of lignification processes during xylem development and compression wood formation, which are key adaptive traits specific to gymnosperms. Despite differences in family size and phylogenetic distribution, PmaLAC proteins exhibit a high degree of structural conservation, showing strong similarity in domain architecture to LAC proteins from *P. densiflora* [58]. Most PmaLAC proteins contain the conserved copper-binding motifs essential for laccase activity, which are enriched in key histidine residues and include the conserved His–Cys–His electron transfer site [65], highlighting the evolutionary conservation of LAC proteins in gymnosperms.

In terms of duplication events, tandem duplication refers to duplicated genes that are adjacent or closely linked on the same chromosome, usually within 200 kb, whereas segmental duplication refers to duplicated genes located on different chromosomes or distant regions of the same chromosome, often arising from large-scale chromosomal rearrangements or partial genome duplications [54,66]. Collinearity analysis identified 16 syntenic gene pairs involving *PmaLAC* genes, suggesting that large-scale duplication events contributed to the evolution of this gene family. Further duplication analysis revealed that 43 *PmaLAC* genes originated from tandem duplication events, whereas 20 genes were classified as segmentally duplicated genes based on synteny analysis (Table S15). These results suggest that tandem duplication was the predominant driving force underlying the expansion of the *PmaLAC* gene family. The tendency of duplicated genes to form chromosomal clusters is consistent with expansion patterns reported for *LAC* gene families in other plant species [1]. Tandem duplication has been recognized as an important mechanism underlying the expansion and diversification of gene families associated with secondary metabolism and cell wall biosynthesis [54,66]. In addition, all 16 duplicated gene pairs exhibited Ka/Ks ratios below 1.0 (Table S16); they have been subjected to strong purifying selection during evolution [67]. This pattern suggests that duplicated *PmaLAC* genes have remained evolutionarily conserved following duplication, which is consistent with observations in other woody plant species [57].

RNA-seq analysis further revealed that *PmaLAC* genes exhibit pronounced tissue-specific expression patterns across the six sampled tissue types. Except for two genes with undetectable expression, the remaining 76 *PmaLAC* genes displayed diverse expression profiles that could be classified into eight distinct types, suggesting functional divergence among family members. According to the duplication–degeneration–complementation (DDC) model, divergence in regulatory regions represents a key mechanism underlying the long-term retention of duplicated genes [68]. Combined with phylogenetic analysis, members within the same subfamily generally exhibit similar expression patterns, suggesting potential coordinated regulatory relationships among them. This is consistent with our findings, which indicate that a single miR397 member can target multiple *PmaLAC* genes. Consistent with this model, cis-acting element analysis showed that *PmaLAC* gene promoter regions are enriched with regulatory elements associated with hormone responsiveness, stress responses, and developmental regulation. Variation in the composition of these cis-elements may contribute to the observed expression diversity, a phenomenon that has also been reported for *LAC* gene families in other plant species, such as *Sorghum bicolor* (sorghum) [41,69]. Collectively, these results suggest that *PmaLAC* gene family members may participate in the fine-tuned regulation of lignin deposition across different tissues through regulatory-level diversification.

miRNA-mediated post-transcriptional regulation represents an important layer of gene expression control in plants, playing critical roles in growth, development, and stress responses [70,71,72]. Gymnosperm genomes are generally characterized by exceptionally large genome sizes and extensive historical gene duplication and retention events [73]. In the present study, a total of 34 miR397 family members were identified in *P. massoniana* based on degradome-supported miRNA datasets, which is considerably higher than the numbers reported in *A. thaliana* (2 members) [74], *O. sativa* (2 members) [75], and *P. trichocarpa* (3 members) [76]. A similar expansion pattern was also observed in *Picea abies* (Norway Spruce) [77], in which 21 miR397 members were identified. These results suggest that the miR397 family has undergone remarkable expansion in conifer species. Such expansion may be associated with the exceptionally large genomes and extensive gene duplication and retention events that characterize gymnosperms [78]. Interestingly, although the precursor sequences of these miR397 members are highly diverse, several distinct precursors generate identical or highly similar mature miR397 sequences, implying that multiple duplicated *MIR397* loci have been retained during evolution while preserving their regulatory functions [54]. Therefore, the expanded miR397 repertoire in conifers is likely attributable to historical duplication events and genome characteristics rather than functional diversification at the mature miRNA level [66]. Moreover, some precursor sequences may represent highly similar duplicated copies that have diverged in their flanking regions while maintaining conserved mature miRNA sequences. This feature may provide a mechanism for coordinated regulation of a large number of target genes and contribute to the complex control of lignin biosynthesis and secondary cell wall formation in woody gymnosperms [78].

miR397 has been widely recognized as a conserved regulator of *LAC* genes in diverse plant species, where it plays important roles in lignin biosynthesis and secondary cell wall formation [11,57]. In the present study, 43 *PmaLAC* genes were predicted to be targeted by miR397, and the target sites exhibited a high degree of sequence conservation, suggesting that miR397-mediated regulation has been largely retained during the evolution of the *PmaLAC* gene family. Notably, this regulatory pattern indicates a complex many-to-one and one-to-many interaction network, in which multiple miR397 members may collectively target a subset of *PmaLAC* genes, while individual miR397 variants can also regulate multiple *PmaLAC* family members, thereby forming a highly interconnected post-transcriptional regulatory module. Furthermore, the interaction between pma-miR397c-9 and *PmaLAC31* was supported by degradome sequencing and experimentally validated through transient expression and dual-luciferase assays, providing direct evidence for miRNA-guided regulation of a *LAC* gene in *P. massoniana*. Considering the central role of *LAC* in lignin polymerization, this miR397–*PmaLAC* regulatory network may contribute to the fine-tuning of lignin biosynthesis and wood formation in gymnosperms [79]. The extensive targeting of *PmaLAC* genes by miR397 may facilitate coordinated post-transcriptional regulation of lignification-related processes during secondary xylem development [56]. These findings suggest that the miR397–*LAC* regulatory module is evolutionarily conserved in conifers and may represent an important regulatory layer controlling wood formation [57].

## 5. Conclusions

In this study, a comprehensive genome-wide analysis of the *LAC* gene family was conducted in *P. massoniana*, leading to the identification of 78 *PmaLAC* genes. Phylogenetic, structural, and duplication analyses indicated that tandem duplication was the primary driver of *PmaLAC* family expansion, contributing to the diversification of *LAC* genes in this conifer species. Expression profiling revealed distinct tissue-specific expression patterns, suggesting functional divergence among *PmaLAC* members during growth and development.

Furthermore, miR397 family members were predicted to target a substantial proportion of *PmaLAC* genes, highlighting a potentially conserved post-transcriptional regulatory mechanism. Notably, degradome sequencing, transient expression assays, STTM-mediated inhibition, and dual-luciferase analyses collectively demonstrated that pma-miR397c-9 directly regulates *PmaLAC31* through cleavage-mediated transcript repression. This finding provides evidence for miR397-mediated regulation of a *LAC* gene in *P. massoniana* and supports the conservation of the miR397–*LAC* regulatory module in gymnosperms.

Overall, our results provide new insights into the evolution, expression, and post-transcriptional regulation of the *PmaLAC* gene family, establishing a valuable foundation for future studies on lignification, secondary xylem development, and wood formation in conifers.

## Data Availability

All data generated or analyzed in this study are provided within this published article and its Supplementary Materials. The raw datasets supporting the reported findings are available from the corresponding author upon reasonable request.

## Supplementary Materials

The following supporting information can be downloaded at: https://www.mdpi.com/article/10.3390/plants15132032/s1.
