# Supplementary material for: Genome-Wide Identification, Expression Profiling, and microRNA397-Mediated Regulation of Laccase Genes in Pinus massoniana
Source: Plants (Basel). 2026 Jun 30;15(13):2032. doi: 10.3390/plants15132032 (PMC13363782; doi:10.3390/plants15132032)
Supplement: Supplementary file 1 [file plants-15-02032-s001.zip › Supplementary Figures.pdf]

Supplementary Figure

Figure S1 Conserved motifs and gene structures of the *PmaLAC* family in *Pinus massoniana*

Figure S2 Chromosomal distribution of genes

Figure.S3 Expression patterns of *PmaLAC* genes in different tissues

Figure S4 Phylogenetic and sequence alignment analysis of the miR397 family in *Pinus massoniana*

Figure S5 Heatmap of miR397 family expression based on TPM values

Figure S6 Cis-acting regulatory elements in the promoter sequences of the miR397 family in *Pinus massoniana*

Figure S7 Interaction network between miR397 and *PmaLAC* genes

Figure S8 t-plot of degradome-predicted miRNA targets (category ≤ 2)

Figure S9 Schematic diagram of STTM397c-9 sequence

Figure S10 Plasmid map of the pGreenII 62SK vector

Figure S11 Plasmid map of the pGreenII 0800-miRNA vector

Figure S12 Plasmid map of the pJIT166 vector

Figure S13 GO enrichment analysis of the LAC gene family in *Pinus massoniana*

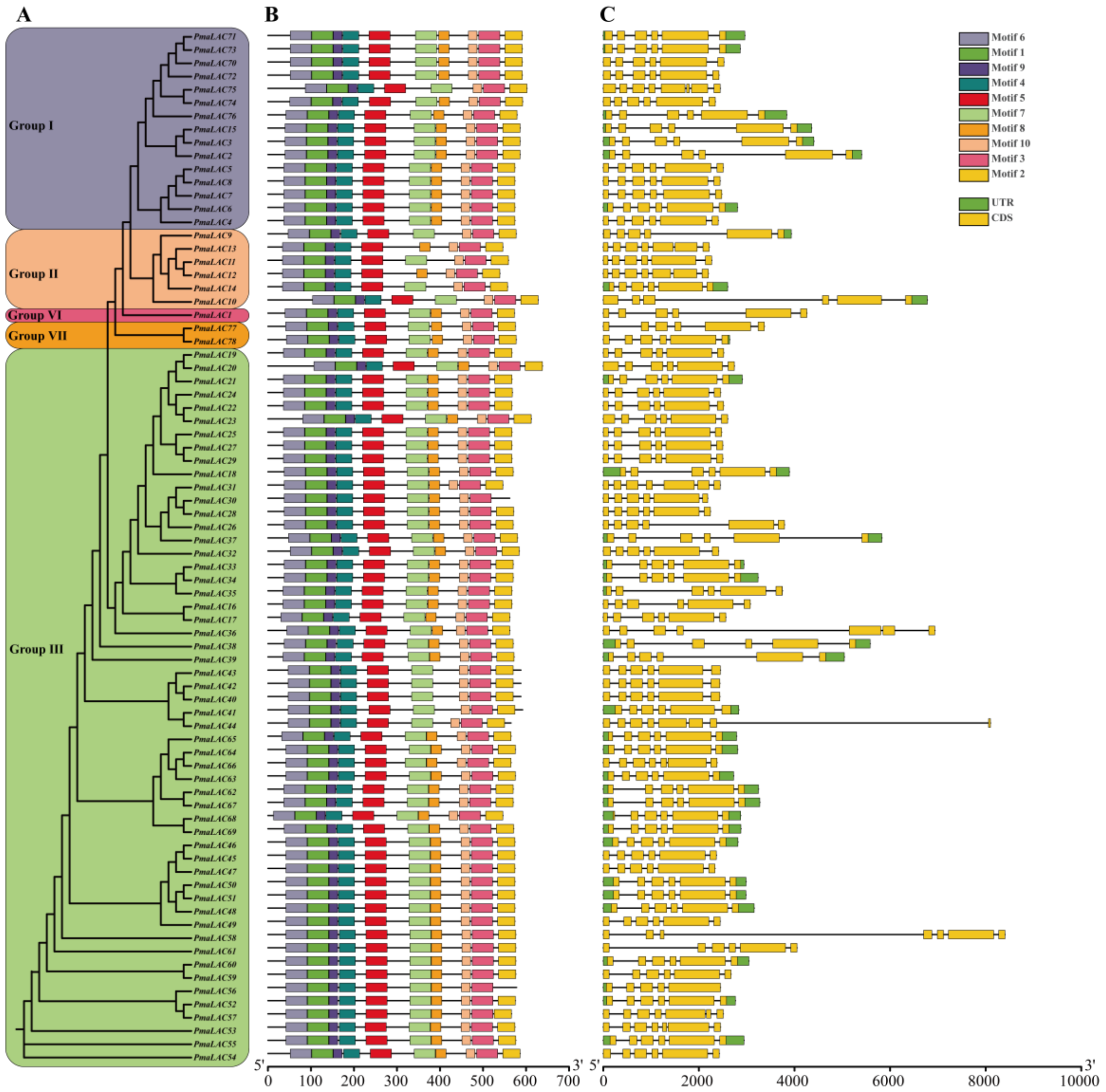

Figure S1. Conserved motifs and gene structures of the *PmaLAC* family in *Pinus massoniana*.(A) Phylogenetic tree of the PmaLAC gene family. Members of different subfamilies are indicated by different colors.(B) Conserved motif analysis of PmaLAC proteins. Lines represent protein sequences, and colored boxes represent distinct conserved motifs. Different colors correspond to different motif types.(C) Gene structure of *PmaLAC* genes. Green boxes represent UTR regions, and yellow boxes represent CDS regions.

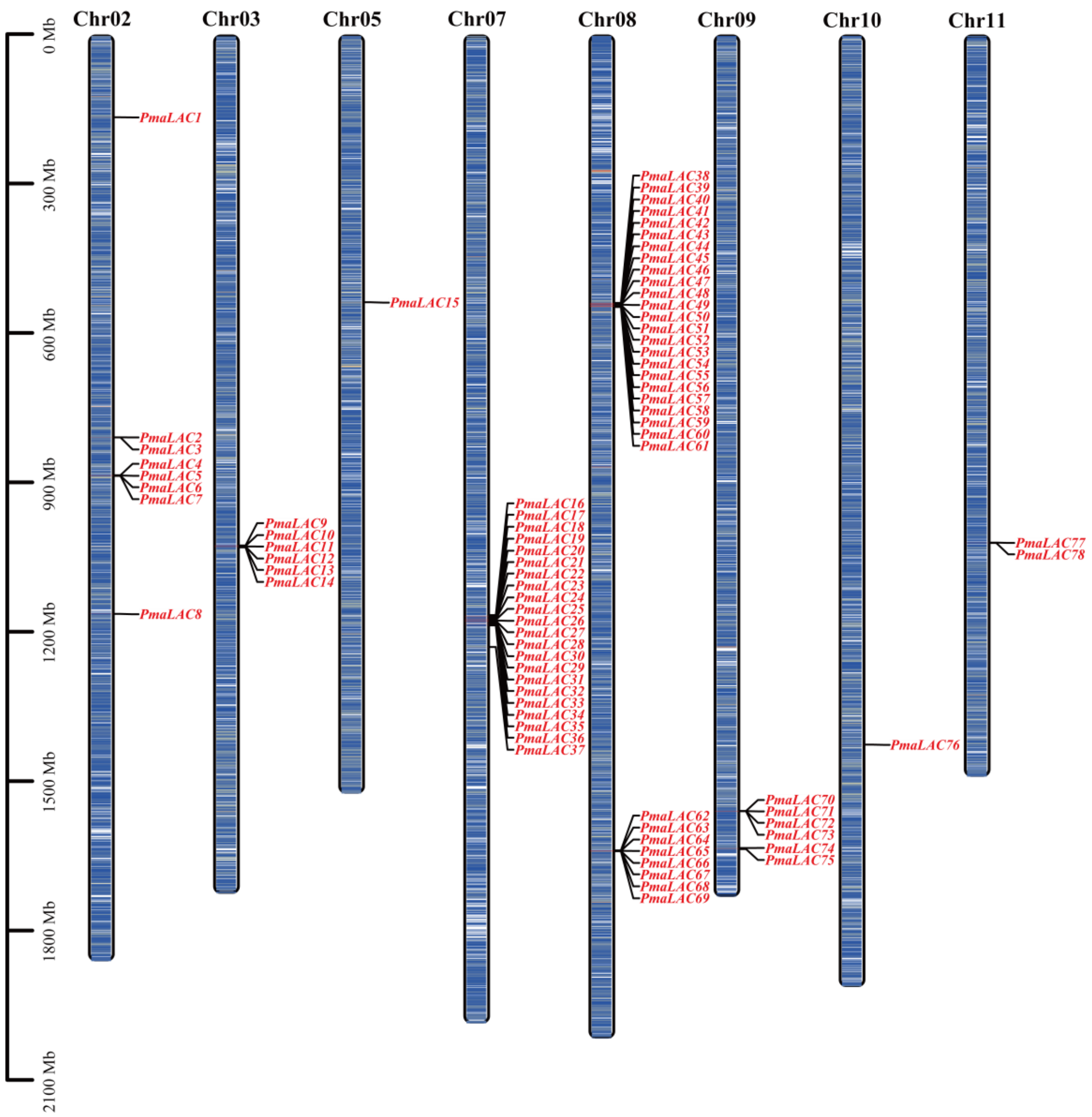

Figure S2. Chromosomal distribution of genes. The left vertical axis represents chromosome length. Black lines indicate the positions of genes on the chromosomes, and a blue-to-white gradient represents gene density along the chromosomes, with darker blue indicating regions containing a higher number of genes.

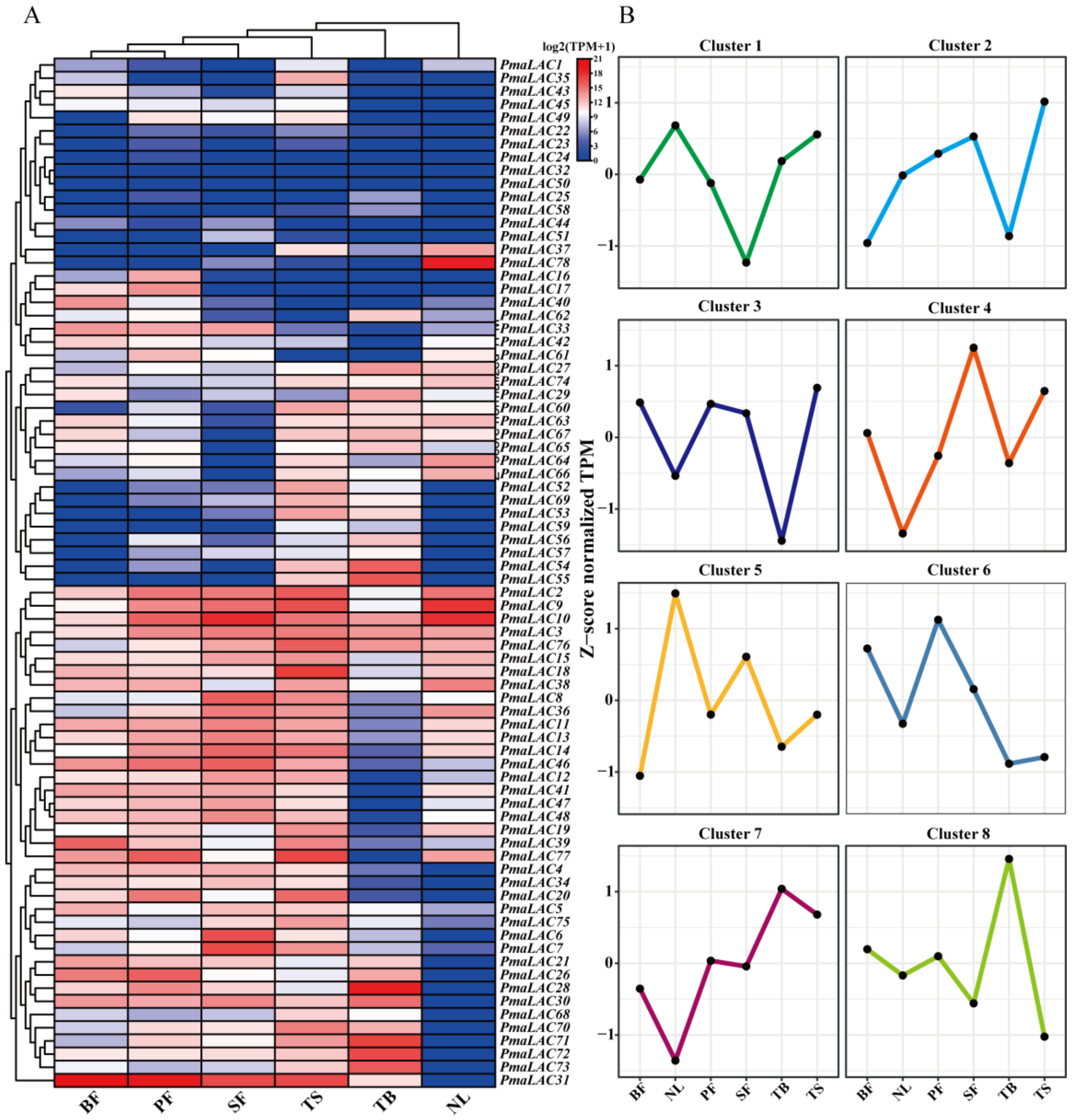

Figure S3. Expression patterns of *PmaLAC* genes in different tissues. (A) Heatmap of *PmaLAC* gene expression across different tissues. Red indicates high expression levels, while blue indicates low expression levels. Genes were hierarchically clustered on the left to show expression similarity. PF, female strobili; SF, male strobili; BF, immature cones (12-month-old); NL, one-year-old needles; TS, young stems (3-month-old); TB, bark tissues (including phloem and developing xylem). (B) Expression clustering analysis based on Z-score normalized TPM values. Genes were grouped into eight expression patterns according to their expression trends across tissues. The lines in the plot represent the overall expression trend of each pattern.

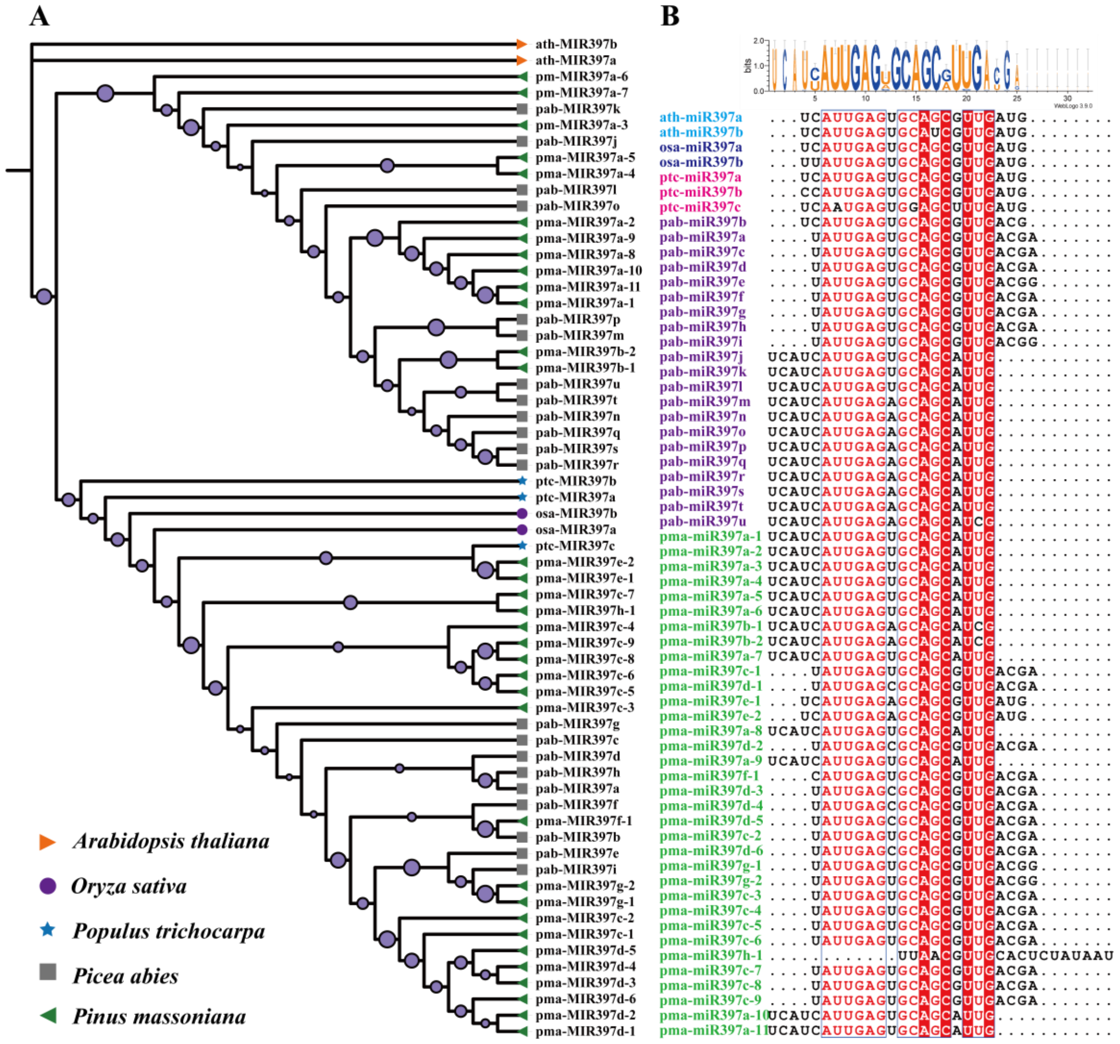

Figure S4. Phylogenetic and sequence alignment analysis of the miR397 family in *Pinus massoniana*.(A) Phylogenetic tree of miR397 precursor sequences from *Pinus massoniana*, *Arabidopsis thaliana*, *Populus trichocarpa*, *Oryza sativa*, and *Picea abies*. Different symbols represent different species.(B) Multiple sequence alignment of mature miR397 sequences from the five species. The WebLogo above illustrates conserved nucleotide positions and their distribution patterns.

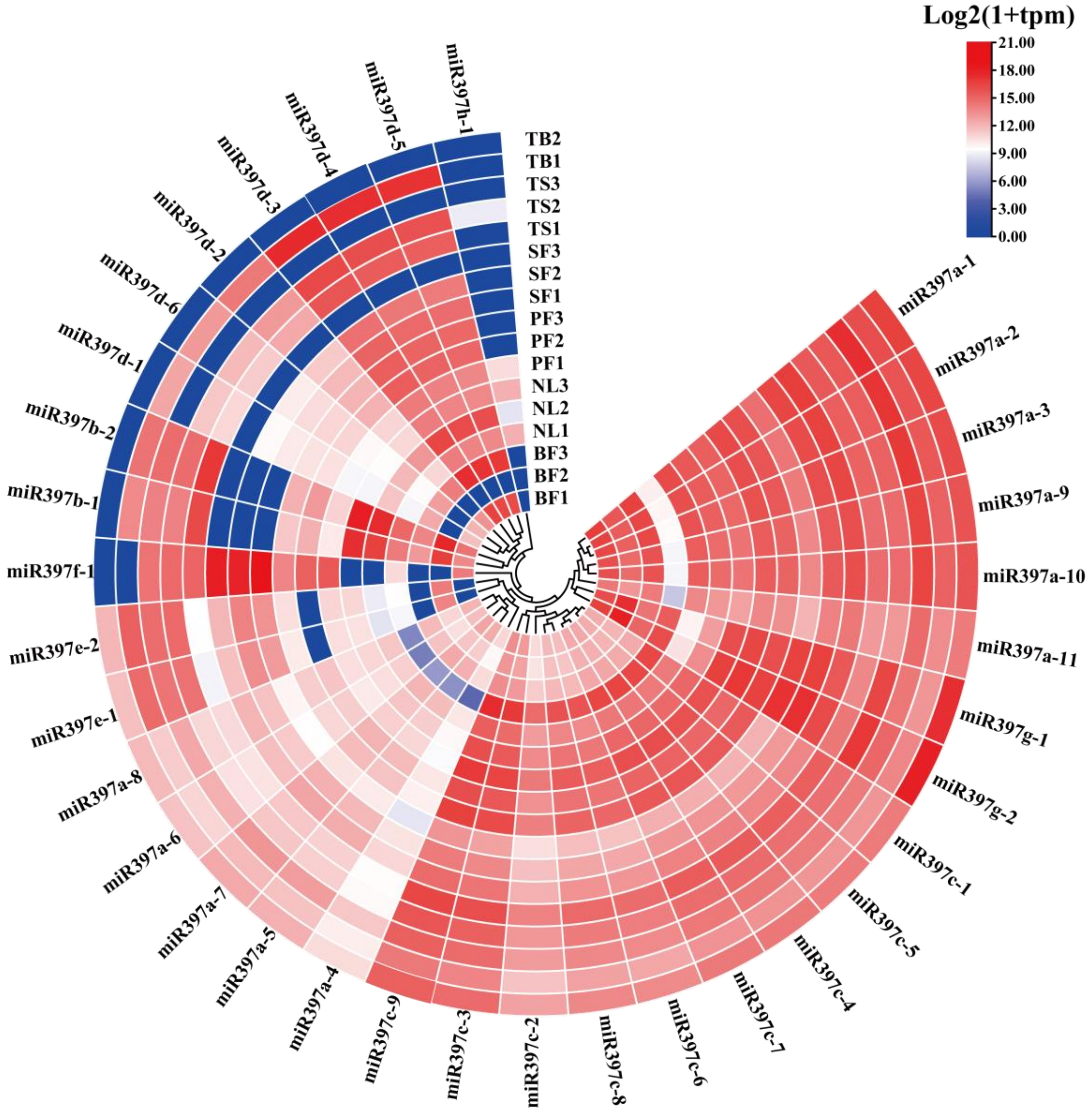

Figure S5. Heatmap of miR397 family expression based on TPM values. The color scale represents expression levels quantified by TPM values; higher expression is indicated by red, while lower expression is indicated by blue.

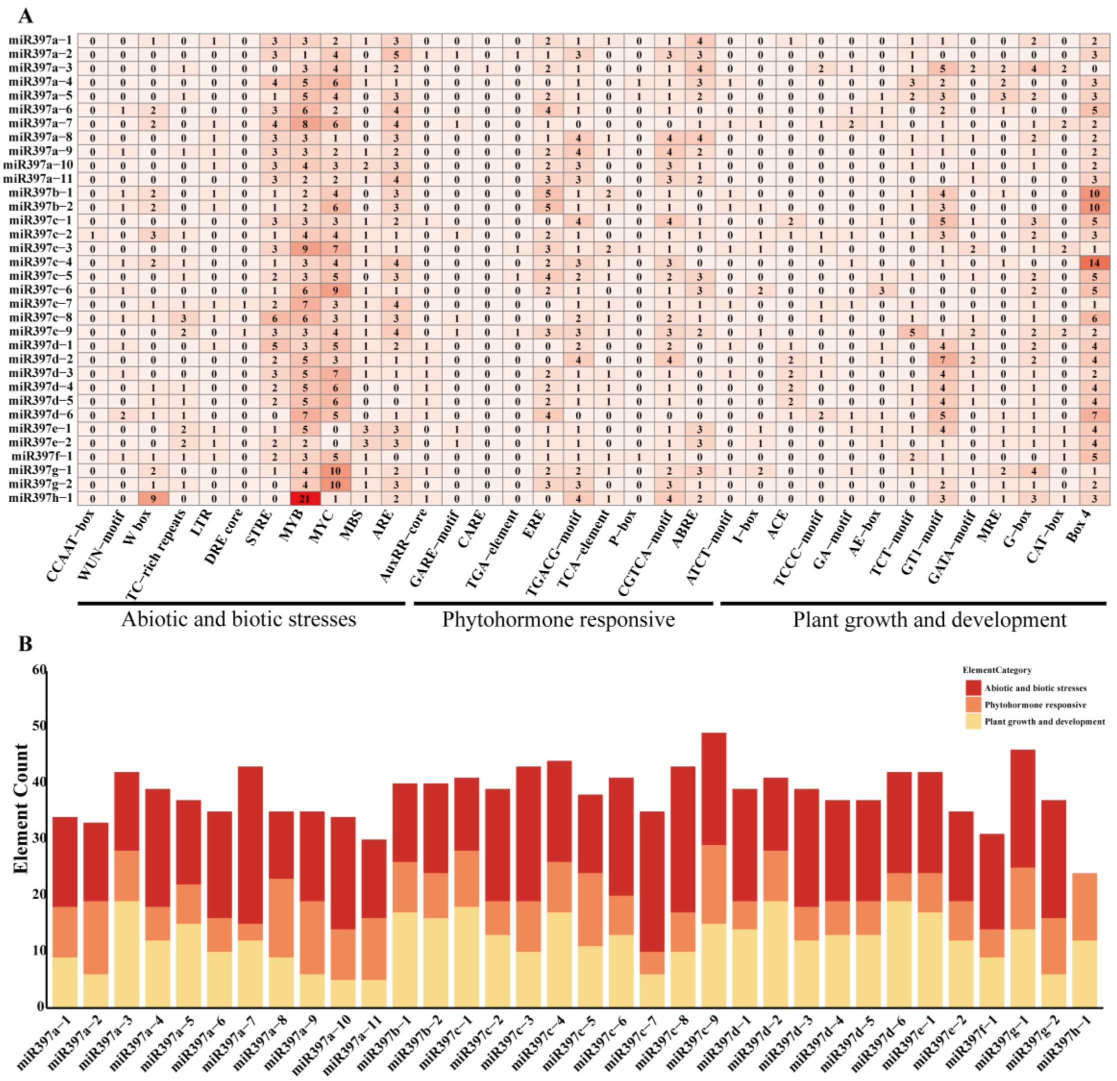

Figure S6. Cis-acting regulatory elements in the promoter sequences of the miR397 family in *Pinus massoniana*.(A) Heatmap showing the types and abundance of cis-acting regulatory elements in the promoters of miR397 family members. The color intensity indicates the number of elements, and the numbers within the heatmap represent copy numbers of each element.(B) Proportional composition of three categories of functional cis-acting elements across different family members. Different colored blocks represent distinct classes of regulatory elements.

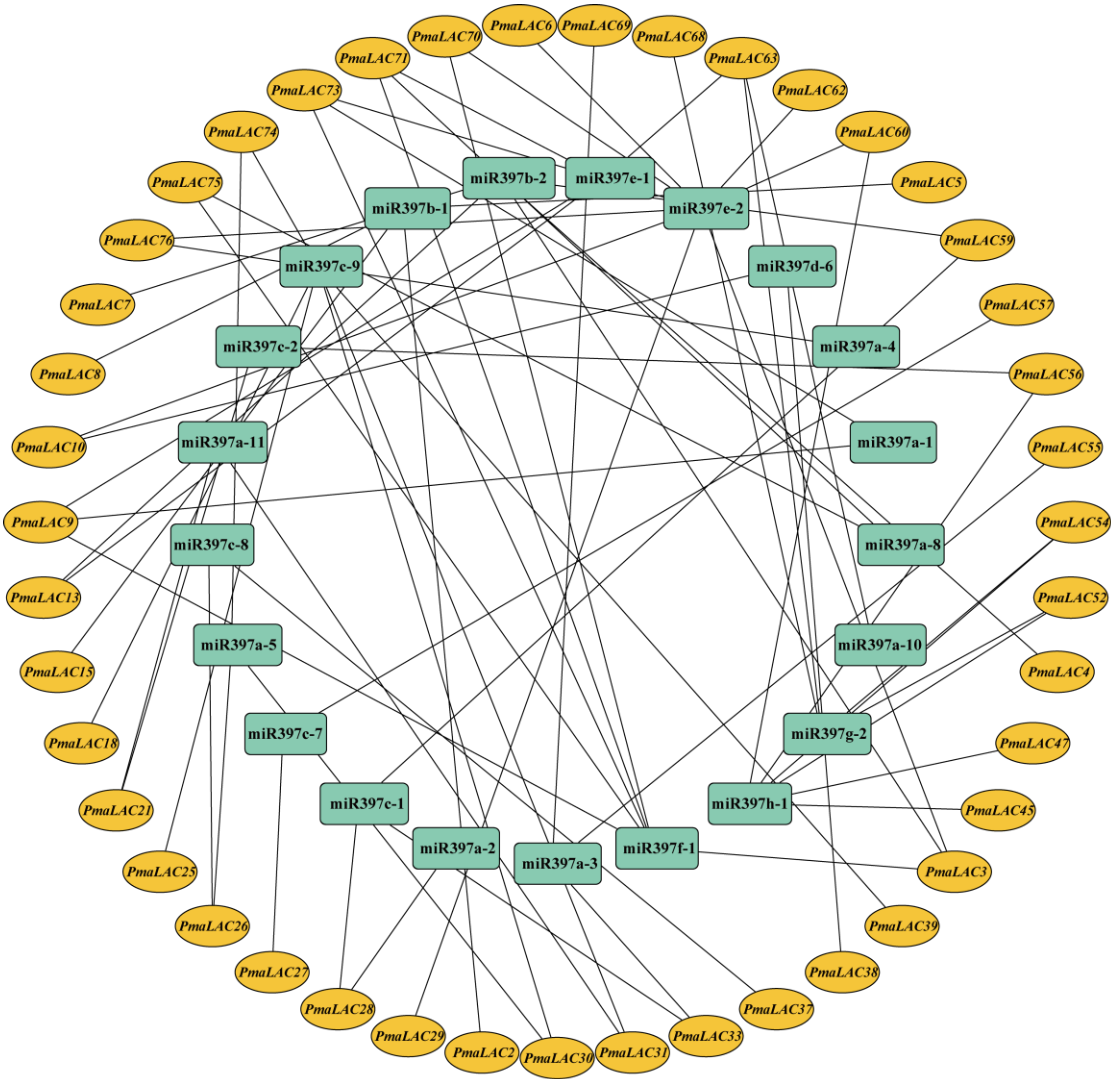

Figure S7. Interaction network between miR397 and *LAC* genes. Based on degradome sequencing data and bioinformatic predictions, miR397 and *PmaLAC* family members are represented by nodes of different shapes. Predicted targeting relationships between the miR397 family and *PmaLAC* genes are indicated by connecting lines. The regulatory interactions are visualized as a network to illustrate potential miRNA–target relationships.

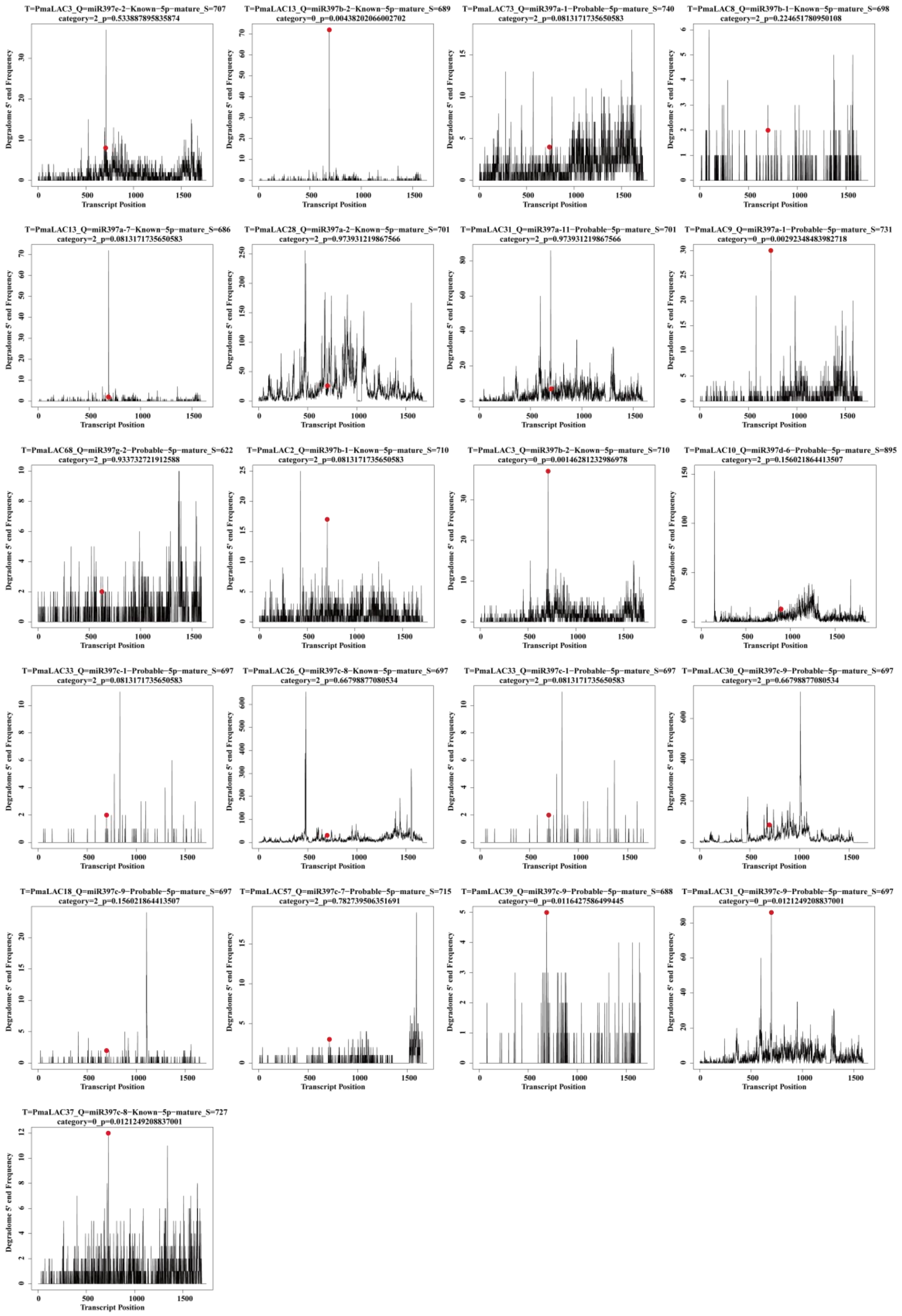

Figure S8. t-plot of degradome-predicted miRNA targets (category ≤ 2). Red circles indicate the predicted target sites.

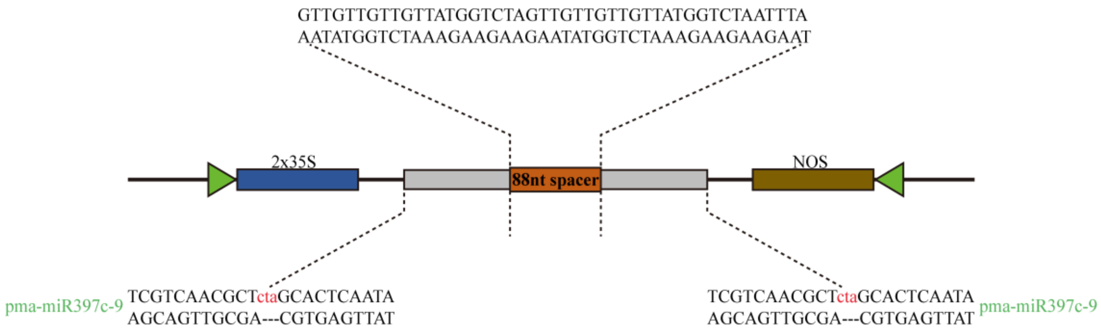

Figure S9. Schematic diagram of STTM397c-9 sequence

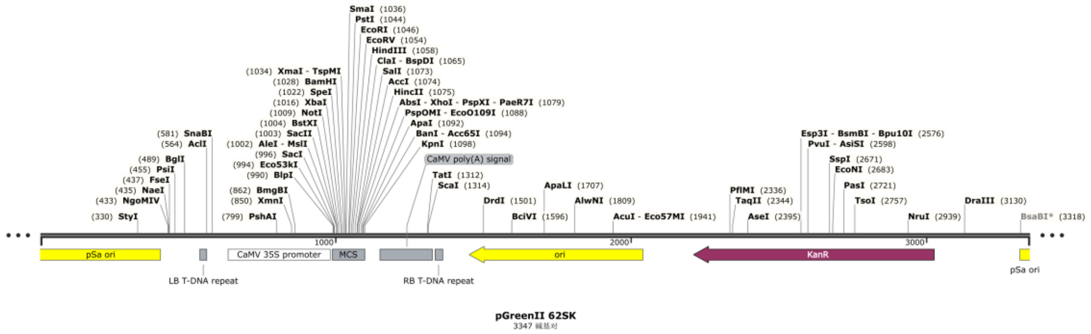

Figure S10. Plasmid map of the pGreenII 62SK vector

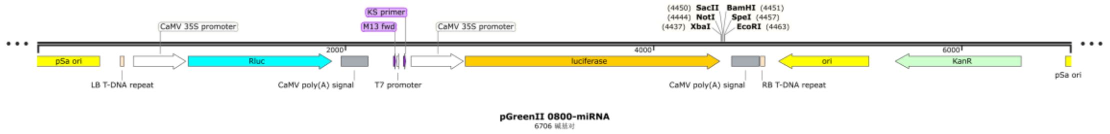

Figure S11. Plasmid map of the pGreenII 0800-miRNA vector

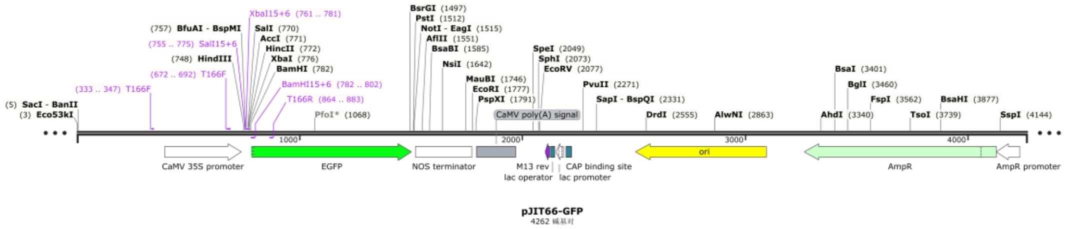

Figure S12. Plasmid map of the pJIT166 vector

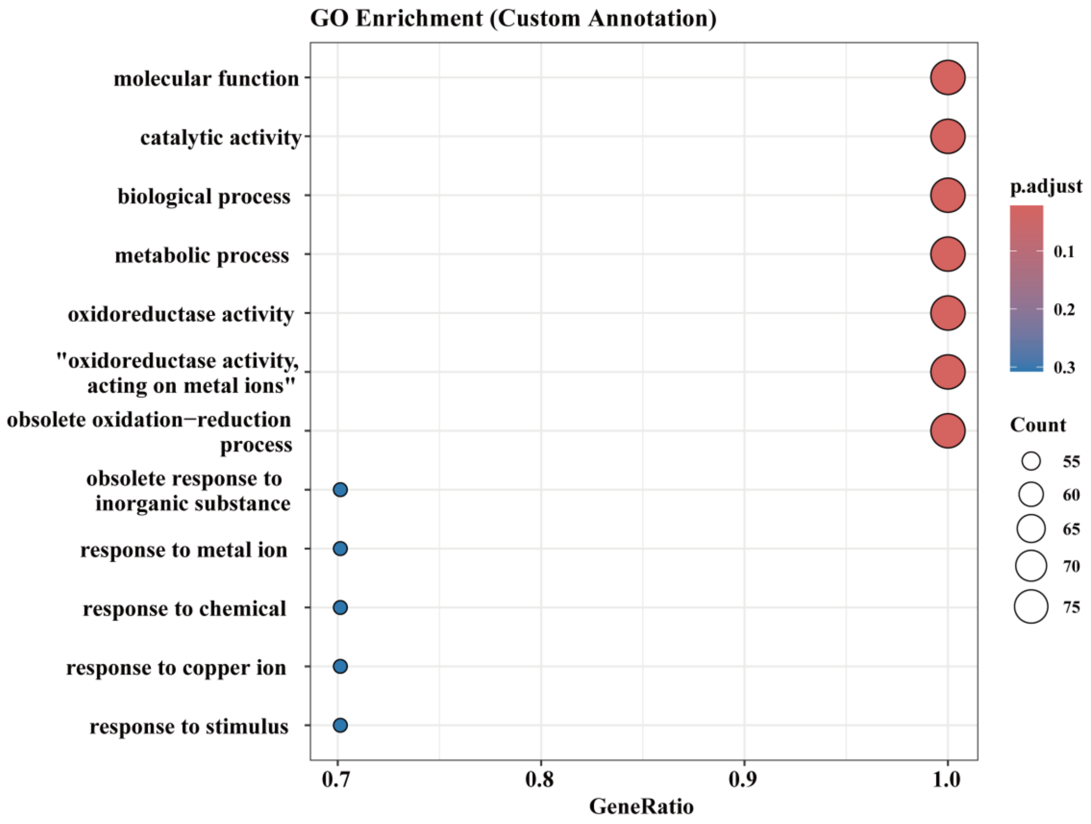

Figure S13. GO enrichment analysis of the *LAC* gene family in *Pinus massoniana*. The size of the bubbles represents the number of enriched genes, with larger bubbles indicating a higher number of enriched genes. The color gradient from blue to red represents the level of confidence, where a deeper red color indicates higher confidence.
