# Supplementary material for: Genome-Wide Identification, Expression Profiling, and microRNA397-Mediated Regulation of Laccase Genes in Pinus massoniana
Source: Plants (Basel). 2026 Jun 30;15(13):2032. doi: 10.3390/plants15132032 (PMC13363782; doi:10.3390/plants15132032)
Supplement: Supplementary file 1 [file plants-15-02032-s001.zip › Supplementary Method.pdf]

Genome re-annotation pipeline of *Pinus massoniana*

Re-annotation workflow

To improve the accuracy of gene models, the *Pinus massoniana* genome was re-annotated by integrating transcriptomic and protein homology evidence. Briefly, repetitive sequences were first identified and masked, followed by the alignment of RNA-seq reads to the reference genome and transcript assembly. Homologous protein sequences from closely related species were then aligned to the genome to provide additional annotation evidence. Gene models were subsequently predicted by integrating transcriptomic and homology information, and multiple lines of evidence were combined to generate a consensus gene set. Finally, functional annotation was performed for the predicted genes, and the completeness of the re-annotated gene set was evaluated.

Software and tools

The re-annotation pipeline employed RepeatModeler v2.0 and RepeatMasker v4.1 for repeat identification and masking, HISAT2 v2.2.1 for RNA-seq read alignment, StringTie v2.2 for transcript assembly, BLAST+ and Exonerate for protein homology alignment, BRAKER2 (integrating GeneMark-ET and AUGUSTUS) for gene prediction, and EvidenceModeler (EVM) for consensus gene model integration. Functional annotation was conducted using InterProScan v5, eggNOG-mapper v2, and BLASTP against the Swiss-Prot database, while annotation completeness was assessed using BUSCO v5.
